# The Physician’s Visiting List for 1892

**Published:** 1892-01

**Authors:** 


### BOOK NOTICES.

The Physician's Visiting List for 1892. Philadelphia: P. Blakiston, Son & Co., 1012 Walnut Street, Phila., Pa.

The present issue of this visiting list represents the forty-first year of its publication. The best recommendation that can be given it is that it is constantly used by the editor of this journal in his private practice. W. M. J.

